# Therapeutic Efficacy of Botulinum Toxin in Trigeminal Neuralgia

**DOI:** 10.7759/cureus.26856

**Published:** 2022-07-14

**Authors:** Abdul Mueez Alam Kayani, Minollie Suzanne Silva, Maleesha Jayasinghe, Malay Singhal, Snigdha Karnakoti, Samiksha Jain, Rahul Jena

**Affiliations:** 1 Medicine and Surgery, Allama Iqbal Medical College, Lahore, PAK; 2 Medicine and Surgery, Nanjing Medical University, Nanjing, CHN; 3 Medicine, Nanjing Medical University, Galle, LKA; 4 Internal Medicine, Mahatma Gandhi Memorial Medical College, Indore, IND; 5 Medicine, Malla Reddy Institute of Medical Sciences, Hyderabad, IND; 6 Medicine, Guntur Medical College, Guntur, IND; 7 Medicine, Bharati Vidyapeeth Medical College/Bharati Hospital, Pune, IND

**Keywords:** therapeutic interventions, botulinum injection, trigeminal nerve, secondary trigeminal neuralgia, botulinum toxin-a

## Abstract

Trigeminal neuralgia (TN) is a unilateral, paroxysmal, sharp, shooting, or jabbing pain that occurs in the trigeminal nerve divisions, including the ophthalmic (V1), maxillary (V2), and mandibular (V3) nerves. Typically, an episode is triggered by anything touching the face or teeth. TN is a clinical diagnosis with no specific diagnostic test; it is determined by the patient's medical history and pain description. Imaging is necessary to exclude secondary causes. The precise reason for TN is uncertain, but it is commonly believed to result from vascular compression of the trigeminal nerve root, typically near its origin in the pons. There are numerous surgical and medical treatment options available. The most frequently applied medical treatment therapies are carbamazepine and oxcarbazepine. Surgical alternatives are reserved for patients who do not respond to medical treatment. Botulinum toxin A (BTX-A) has emerged as a novel and promising alternative to surgery for individuals whose pain is unresponsive to medication. Multiple studies have established the safety and usefulness of BTX-A in treating TN, with the most significant benefits occurring between six weeks and three months after the surgery. This article reviews various studies published in the last 10 years regarding the therapeutic use of BTX-A in TN. These studies include various observational, clinical, pilot, and animal studies.

## Introduction and background

The International Headache Society defines trigeminal neuralgia (TN) as a disease characterized by recurrent unilateral, brief electric shock-like pains that are abrupt in onset and termination, limited to the distribution of one or more divisions of the trigeminal nerve, and triggered by innocuous stimuli [1]. Idiopathic TN most frequently affects people between the age of 40 and 60, with females involved more often than males [2]. Diverse epidemiological studies indicate that the global incidence of TN ranges from four to 28.9 per 100,000 individuals [2,3]. However, the precise cause of TN remains uncertain [4]. Most cases of TN are caused by vascular compression of the nerve root, typically within a few millimeters of the pons. Approximately, 80%-90% of idiopathic TN cases are caused by vascular compression by an artery or nerve loop. Other compressive causes include acoustic neuromas and meningiomas of the posterior fossa. Demyelination of sensory fibers within the nerve root, root entrance, or brain stem itself is frequently accompanied by nerve compression. This demyelination causes ectopic firing along the path of the trigeminal nerve [5].

The three types of TN are classical, secondary, and idiopathic. The classical type, which is the most prevalent, accounts for 75% of all cases of TN. It is diagnosed when trigeminal neurovascular compression results in morphological changes ipsilateral to the side of the compression, demonstrated with magnetic resonance imaging or surgery [6]. The second most prevalent form accounting for 15% of all cases, the secondary type, is diagnosed when a neurological disease known to cause TN is present. The idiopathic type is the least common type and is diagnosed when no apparent cause can be found [7].

The diagnosis of TN is based solely on the clinical presentation as no particular diagnostic test exists. TN is characterized by a sharp, shooting, stabbing pain that is unilateral, abrupt in onset, and can persist for a fraction of a second to up to two minutes. These paroxysmal episodes are frequently precipitated by non-noxious stimuli, such as tooth or facial contact [8].

**Figure 1:**
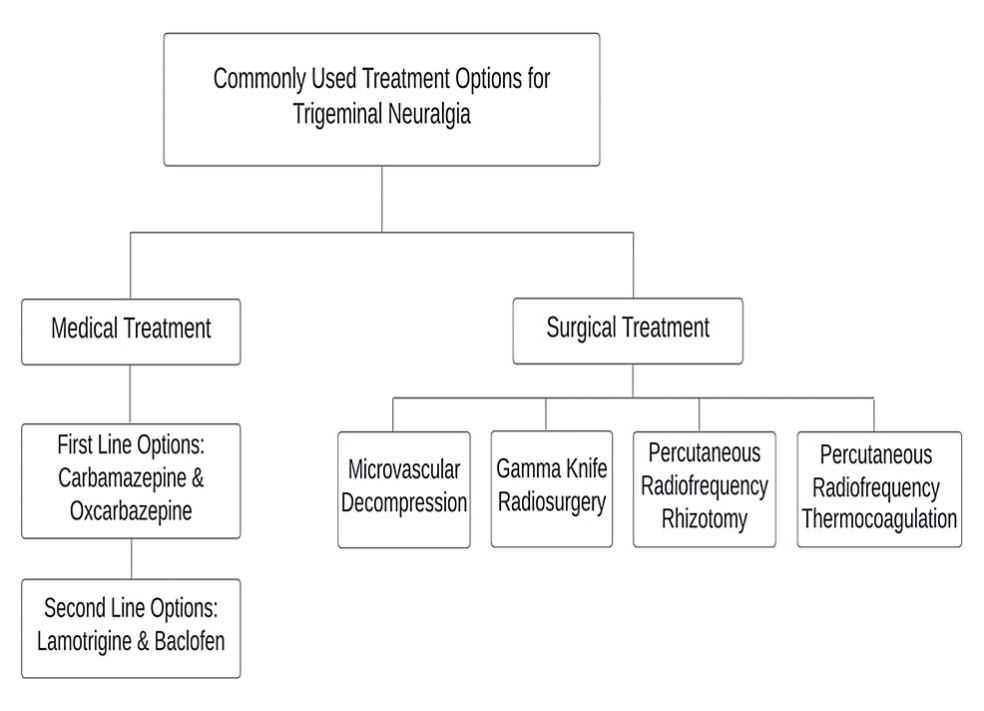
Surgical and medical therapy options for trigeminal neuralgia (TN) Image credit: Dr. Abdul Mueez Alam Kayani.

There are numerous surgical and medical treatment options available. Carbamazepine and oxcarbazepine are first-line therapy options, whereas lamotrigine and baclofen are second-line alternatives. However, medical treatments have their associated adverse effects [9]. Guidelines recommend surgical therapies for patients whose symptoms are refractory to medications [10]. Surgical options include microvascular decompression, Gamma Knife stereotactic radiosurgery, percutaneous radiofrequency rhizotomy, and percutaneous radiofrequency thermocoagulation [11-14]. Surgical therapeutic approaches may result in severe, incurable consequences that are more disabling than TN itself [15]. The failure rates and complications observed with standard medical and surgical treatment methods necessitate a close examination of botulinum toxin A (BTX-A) as a viable alternative [16]. The commonly used medical and surgical treatment options are depicted in Figure 1.

**Figure 2:**
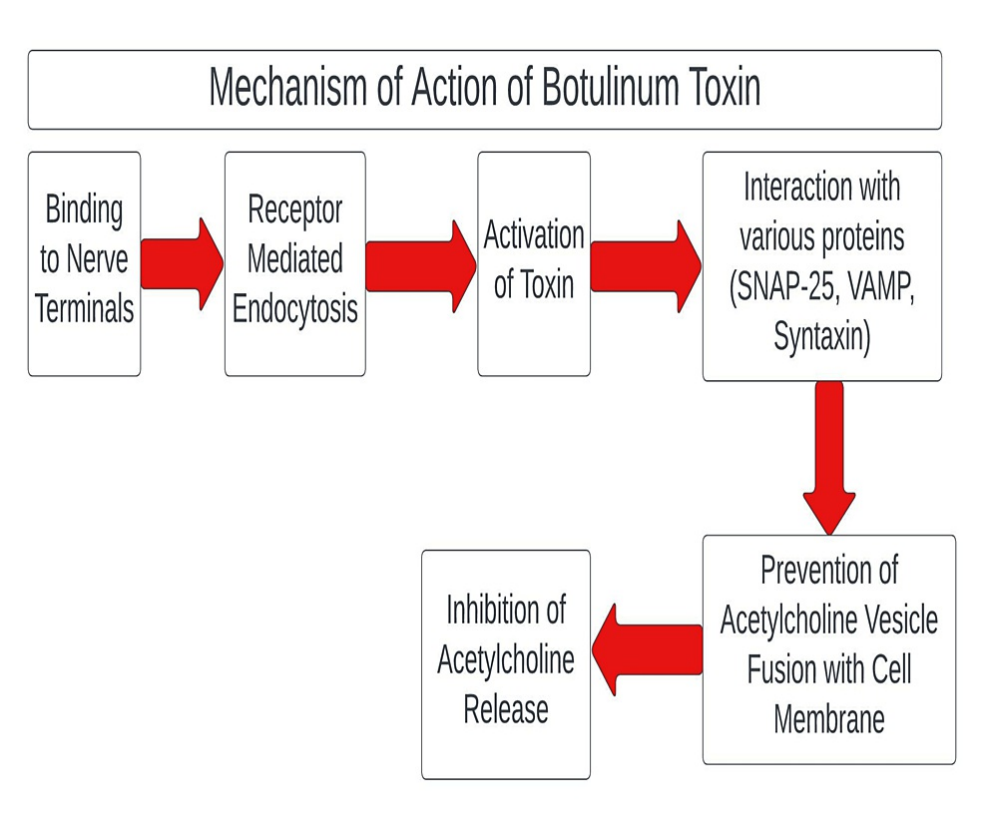
Mechanism of action of botulinum toxin (BTX-A) Image credit: Dr. Abdul Mueez Alam Kayani.

The Food and Drug Administration (FDA) initially approved botulinum toxin to treat strabismus [17]. BTX-A is a naturally occurring neurotoxin produced by *Clostridium botulinum*, an anaerobic gram-positive bacteria. There are eight distinct antigenic types A-G with type A having the most potent effect. Botulinum toxin primarily acts by preventing the release of acetylcholine at the neuromuscular junction. The toxin prevents the release of acetylcholine by cleaving critical proteins involved in its release [18]. The mechanism of action of BTX-A is shown in Figure 2.

**Figure 3:**
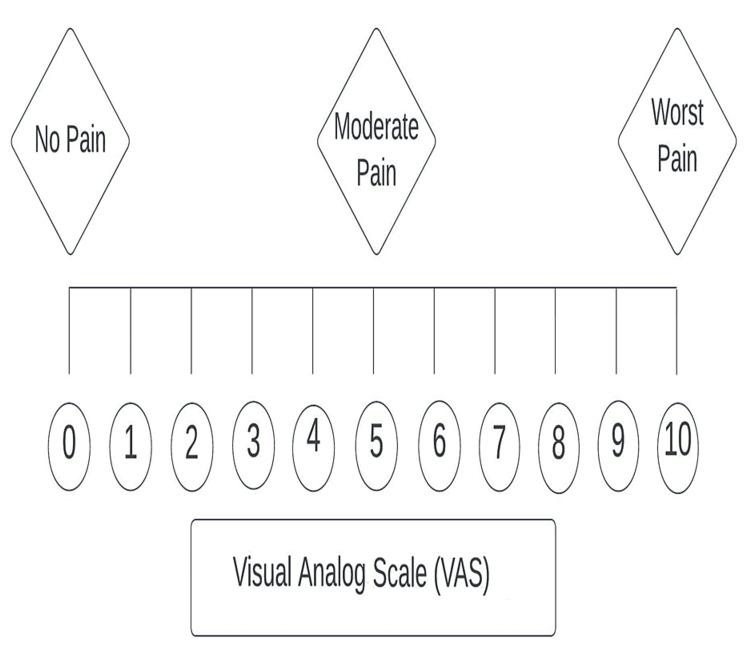
Visual analog scale (VAS) to determine the pain severity and intensity Image credit: Dr. Abdul Mueez Alam Kayani.

The efficacy of BTX-A in TN is usually measured on a visual analog scale (VAS), which is commonly used in epidemiological and clinical research to measure the frequency and intensity of symptoms. The VAS is depicted in Figure 3.

Even though the initial results regarding the use of BTX-A in TN seem promising, various factors limit its widespread use in real-world clinical practice. The clinical trials conducted so far have a small sample size limiting the clinical usefulness of the conclusions drawn regarding its use.The patients selected for studies have intractable TN restricting the efficacy of conclusions that we can draw from these studies regarding the use of BTX-A as a first-line treatment option for TN. There is a shortage of evidence regarding the optimal dose, route, depth of injection, onset of action, and period of effectiveness of BTX-A. Information regarding the safety of BTX-A in pregnancy, nursing, and patients planning on becoming pregnant is unknown. There is also insufficient evidence regarding the clinical use of BTX-A on TN in patients with neuromuscular disorders and medically unstable patients. Patients taking medications that affect neuromuscular junctions, e.g., aminoglycosides and quinine, were excluded from the studies due to the possible interaction of these medications, limiting the clinical usefulness of conclusions drawn from these studies. Information regarding the long-term effectiveness of BTX-A in patients with TN remains to be known since most studies conducted so far have had a short follow-up period. There is a requirement for well-designed randomized, double-blind placebo-controlled trials to overcome these limitations. Even though there has not been a report in the clinical trials assessing the efficacy of BTX-A in TN, there is a concern regarding the risk of possible hypersensitivity reaction with BTX-A administration. There have been reports of hypersensitivity reactions and long-term adverse effects with BTX-A administration when used for cosmetic and therapeutic purposes, necessitating a much more cautious approach toward the therapeutic use of BTX-A in TN [19,20].

## Review

Method

Databases such as PubMed, PubMed Central (PMC), ResearchGate, and Google Scholar were searched. They were extensively searched using relevant keywords and medical subject heading (MeSH) to identify all the relevant articles that discuss the role of BTX-A in the management of TN. Both the MeSH terms "botulinum toxin" and "trigeminal neuralgia" were included in the search. We included articles from 2012 to 2022 and included reviews, clinical trials, pilot studies, animal studies, and retrospective studies. We included only those studies for which complete text was available for free and was in English.

Results

An advanced MeSH on PubMed and Google Scholar yielded 1,143 articles at first. All papers not directly related to the research topic were manually omitted, leaving 18 articles for our review.

Discussion

Following are the findings regarding the clinical use of botulinum toxin in the management of TN.

Observational Studies/Clinical Trials

An open-labeled clinical trial with 27 patients treated with maxillary/mandibular injections of BTX-A showed significant improvement in pain and attack frequency in the first week, second month, and sixth month after treatment. In the second and sixth months, 74.1% and 88.9% of patients, respectively, responded to the treatment. At the sixth-month interval, 44% of the patients did not experience pain attacks. The severity and frequency of pain attacks decreased after each injection administration. Two patients remained recurrence-free after two years of treatment with BTX-A. The success rate of 88.9% achieved with this different injection technique was slightly higher than that attained from other injection methods [21]. An open retrospective study involving 152 patients showed an 89.4% effective rate within two weeks with botulinum toxin injection, with more than 50% reduction of pain on the VAS required for the treatment to be deemed effective. The study also demonstrated long-term efficacy with no significant increase in pain on the VAS at a six-month follow-up. Female sex, short-term disease course, and higher injection dose (more than 70 units) were associated with lower long-term VAS, although these results were not statistically significant. Patients who had received short-term medium (50-70 units)- or high (more than 70 units)-dose injections were more likely to be cured [22]. A clinical trial with 88 patients receiving BTX-A demonstrated improvement, with 92.1% of the patients showing improvement at the one-month interval and 100% at the second-month interval. The effectiveness decreased after the third month of treatment to 38.6% in the 14th month. More than 90% of patients with effective treatment reported that their symptoms felt "improved" or "very much improved," indicating improvement in the TN symptoms, quality of life, emotional function, and the burden of side effects [23].

A randomized, single-blind controlled trial conducted on 20 patients with intractable TN showed a significant reduction in their pain assessed using the VAS with a decrease of 6.5 in the BTX-A group versus 0.3 in the placebo group. There was also a reduced need for acute medications and improved quality of life functioning with BTX-A. No significant relationship was found between total injected dosage and endpoint VAS or pain attack frequency [24]. A randomized, double-blind placebo-controlled trial using 84 patients showed significant improvement in symptoms in patients treated with BTX-A with no difference seen with different doses (25U group versus 75U group). The response rates of 70.4% (25U group) and 86.2% (75U group) were significantly higher than the 32.1% of the placebo group. Improvement in symptoms was reported as early as week 1 of the study, with sustained effects seen until week 8 [25]. An observational study of 22 patients who received BTX-A reported a reduction in the mean pain scores from 20.4% to 33.1%, with a maximum response seen at 60 days following treatment. No differences were observed with different dosing and injection strategies [26].

A clinical trial assessing the safety and efficacy of BTX-A in the management of older patients showed no difference in patients above 80 years and below 60 years of age, with efficacy and dosages comparable between the two age groups. The VAS scale for the older patient group decreased from a baseline of 8.5 to 4.5 after treatment with BTX-A, while for the younger patient group, the VAS decreased from a baseline of 8.0 to 5.0 after treatment with BTX-A [27]. In another retrospective cohort study conducted with 104 patients, a higher treatment success rate of 83.7% was demonstrated with BTX-A, with a higher success rate achieved in patients above 50 years old. However, no significant difference was seen in younger and older patient groups regarding pain recurrence, time to take effect, and time to achieve peak effect [28]. No study reported any significant systemic side effect with BTX-A administration, with transient facial asymmetry reported as the most common side effect [21-28]. Observational studies and clinical trials in our study are summarized in Table 1.

**Table 1:** A compilation of observational and clinical studies conducted to determine the efficacy of BTX-A in trigeminal neuralgia (TN) VAS: Visual analog scale.

Authors	Study type	Number of patients	Effective rate	Adverse events	Conclusion	Limitation of the study
Türk et al. [21]	Open-label	27	74.1% in the second month and 88.9% in the sixth month	Short-term facial asymmetry and masseter weakness on the injection sides	Highly effective and safe	Lack of a placebo, open-label study, and small number of patients
Zhang et al. [22]	Retrospective cohort	152	89.4% of the patients reported improvement during the initial six months of follow-up	Short-term facial asymmetry	Highly effective and safe	
Li et al. [23]	Open-label	88	92.1% in the first month and 100% in the second month	Local injection swelling and muscle relaxation	Highly effective and safe	Open-label study
Shehata et al. [24]	Randomized, single-blind, placebo-controlled	20	Pain on the VAS reduced by 6.5 at the third month	Facial asymmetry, hematoma, itching, and pain	Highly effective and safe	Small number of patients
Zhang et al. [25]	Randomized, double-blind, placebo-controlled	84	70.4% for the 25U group and 86.2% for the 75U group	Short-term facial asymmetry and transient edema	Highly effective and safe with similar efficacy seen with low (25U)- and high (75U)-dose groups	
Caldera et al. [26]	Observational	22	20.4%-33.1% reduction in pain scores	No injection-related severe side effects reported	Highly effective and safe in the management of refractory trigeminal neuralgia	
Liu et al. [27]	Randomized controlled trial	43	Reduction of pain on the VAS from 8.5 to 4.5 in old and 8.0 to 5.0 in young patient groups after one month of treatment	Facial weakness in younger patients and whole-body discomfort and facial asymmetry in older patients	Highly effective and safe in treating older patient groups with doses similar to younger patients	No placebo groups and small number of patients in the older patient group
Wu et al. [28]	Retrospective cohort	104	83.7% of patients	Facial asymmetry	Highly effective and safe with a higher efficacy rate for patients aged 50 or more	Retrospective design and single-center study

Pilot Studies

A pilot study with 100 patients assessing the efficacy of single and repeated doses of BTX-A in the management of TN showed no difference in frequency, the time between treatment and effect, VAS, and adverse effects. The single-dose group reported a significantly longer duration of effects. This study indicates no significant advantage of repeat doses with BTX-A when compared with a single dose of BTX-A [29]. A study involving 10 TN patients treated with onabotulinum toxin A in the sphenopalatine ganglion showed that onabotulinum toxin A is safe and efficacious in the management of TN, with two patients going into full remission. Adverse effects were observed in six patients with all of them being mild with the exception of one patient who experienced moderate paralysis of the inferior rectus muscle [30].

Animal Studies

An animal study involving purified BTX-A to treat experimental trigeminal neuropathy in rats demonstrated significant improvement in the head withdrawal threshold as compared to saline-injected rats, demonstrating the possible efficacy of BTX-A in TN. Although the exact mechanism behind the effect of BTX-A is unclear, possible axonal transport is thought to play a role [31]. A study assessing the efficacy of BTX-A through inhibition of microglia toll-like receptor 2 (TLR2)-mediated neuroinflammation in mice demonstrated that BTX-A significantly inhibited microglia activation, thus demonstrating the mechanism behind the efficacy of BTX-A in TN. Subcutaneous unilateral injection of BTX-A in whisker pad attenuated the bilateral mechanical pain hypersensitivity and anxiety-like behaviors [32]. Another study involving rats showed that BTX-A when administered peripherally localized bilaterally in the trigeminal ganglia and demonstrated a role of axonal and hematogenous transport in the therapeutic role of BTX-A. Head withdrawal threshold was restored to baseline bilaterally, after first being decreased with intraperitoneal cisplatin injection, with a unilateral injection of BTX-A in whisker pads [33].

Review Articles

In a randomized, double-blind placebo-controlled study with 42 patients, intradermal/subcutaneous injection of BTX-A resulted in more than 50% reduction of pain in the VAS in 68.18% of patients. In a class IV, open-ended study with eight patients, all patients reported beneficial effects with BTX-A. In a class IV, open-label study with 13 patients, four patients were pain-free, and nine patients reported more than 50% pain reduction on the VAS with BTX-A, which lasted for 60 days. Transcutaneous injection of BTX-A in 11 patients resulted in more than 50% reduction in pain frequency and intensity in eight patients, with beneficial effects lasting for two to four months. In a class IV, open-label study with 12 patients with TN, 10 patients reported significant improvement in their pain on the VAS with BTX-A with effects lasting for 60 days. Similar results were seen in another class IV, open-label study with 15 patients, with all 15 patients reporting an improvement in pain frequency and intensity with BTX-A with effects lasting up to six months. Although the beneficial effects of BTX-A in TN are reported in several case reports and open-label studies including class IV studies, there has only been a single class I study establishing the therapeutic efficacy of BTX-A in TN [34].

In an open-label study with 12 patients, 83% of patients reported pain relief within a few minutes of injection with BTX-A. Higher doses of BTX-A were associated with a faster onset of action, and mixing BTX-A with lidocaine resulted in the prolonged action of lidocaine, helping in the management of acute exacerbations [35]. The use of BTX-A was associated with lower pain scores in patients compared to saline-injected patients, with relief of pain seen in both short and long terms. Injection doses varied, with most patients receiving 100-200U subcutaneously or intradermally. The use of BTX-A was also associated with an improvement in diabetic neuropathic pain, occipital neuralgia, carpal tunnel syndrome, and phantom limb syndrome [36]. The efficacy of BTX-A was demonstrated in a randomized, double-blind placebo-controlled trial where the effective rate was 70.4% (25U), 86.2% (75U) as compared to 32.1% observed for control. A prospective, open, case series demonstrated an improvement in the frequency and severity of pain in all patients treated with BTX-A. In another prospective, open, case series with 12 TN patients, 10 patients reported a reduction in pain on the VAS, with effects lasting greater than two months. The possible underlying mechanism involves inhibition of the release of inflammatory mediators and peripheral neurotransmitters from sensory nerves [37]. In one double-blind randomized controlled trial and five prospective open-labeled studies, the response rate with BTX-A in TN was seen in 70%-100% of patients, and the attack frequency was reduced by 60%-100% at the four-week interval in most studies. In another study, 47% of patients needed no further treatment, and 33% of patients required non-steroidal anti-inflammatory drugs (NSAIDs) to decrease their pain [2].

Limitations

This review is limited to studies in the English language, so we may have missed important studies that were published in other languages. This review is limited to studies published after 2012, which may have also caused a similar limitation.

## Conclusions

The clinical efficacy of BTX-A in TN possibly stems from the inhibition of the release of inflammatory mediators and peripheral neurotransmitters from sensory nerves. Our review has shown that the studies conducted regarding the clinical use of BTX-A in TN have demonstrated its clinical efficacy, with patients reporting improvement in their pain frequency and intensity. In some cases, long-term benefits with BTX-A are also seen, with patients reporting prolonged pain-free periods. The side effects observed with BTX-A were mild and self-limited in most cases, with no significant systemic side effects reported in most studies. However, several factors limit the clinical use of BTX-A; these include a lack of specific guidelines about optimal dose and injection techniques. Patients included in studies have intractable TN, limiting the usefulness of the conclusions drawn regarding the use of BTX-A as a possible first-line therapy. Safety of BTX-A in patients who are medically unstable, pregnant, or planning to become pregnant remains yet to be determined. There is also a concern regarding the possible drug interaction in patients taking other medications. These concerns necessitate well-designed randomized controlled trials with a large patient sample to fully establish its safety, efficacy, and other parameters, including dosing, route, the onset of action, and effective period. BTX-A can be an effective and viable alternative, especially in patients with intractable TN.
